# 整合素α5β1介导细胞外信号调节激酶信号转导通路对A549细胞生长和侵袭的影响

**DOI:** 10.3779/j.issn.1009-3419.2011.07.02

**Published:** 2011-07-20

**Authors:** 晶 白, 小宁 钟, 海娟 唐, 志义 何, 建全 张, 静敏 邓

**Affiliations:** 530021 南宁，广西医科大学第一附属医院呼吸内科 Department of Respiratory Medicine, the First Affiliated Hospital of Guangxi Medical University, Nanning 530021, China

**Keywords:** 整合素, A549细胞, ERK1/2（细胞外信号调节激酶1/2）, Integrin, A549 cell, ERK1/2 (extracelluar signal-regulated protein kinase 1/2)

## Abstract

**背景与目的:**

近年研究显示整合素α5β1作为整合素家族的重要部分与非小细胞肺癌的转移性、浸润性和低分化趋向密切相关。本研究应用整合素α5/β1 siRNA双链及ERK抑制剂PD98095干预人肺癌细胞A549，探讨整合素α5β1蛋白表达对A549细胞生长和迁移能力的影响及其细胞内信号转导机制。

**方法:**

实验分为未转染组、脂质体组、整合素α5β1 siRNA转染组和PD98095组四组。采用免疫印迹（Western blot）和逆转录-聚合酶链反应（RT-PCR）检测A549中整合素α5β1蛋白和mRNA表达水平，Western blot检测A549中ERK1/2、MMP-9和caspase-3蛋白水平，四甲基偶氮唑盐比色（MT）法和Annexin-V FITC PI双染色法检测A549增殖和凋亡。

**结果:**

整合素α5/β1 siRNA双链在抑制α5/β1蛋白和mRNA表达的同时，可以下调ERK的mRNA和蛋白表达，并抑制其磷酸化水平。整合素α5/β1 siRNA双链和PD98059均可以明显抑制A549细胞的增殖，促进细胞凋亡和细胞内caspase-3蛋白表达，并抑制细胞内MMP-9蛋白表达。

**结论:**

整合素α5β1可能通过介导的ERK信号转导通路参与了A549细胞的增殖和迁移调控。

## Abstract (en)

**Background and objective:**

Recent studies have shown that integrin α5β1 as a core in the integrin family plays an important role in metastasis, invasion and poor difference of non-small cell lung cancer. In this study, A549 cells were cultured and treated with integrin α5β1 small interfering RNA (siRNA) and extracelluar signal-regulated protein kinase (ERK) inhibitor PD98059 to investigate the effect of integrin α5β1 on proliferation and migration of A549 cells and explore its signal transduction mechanism.

**Methods:**

A549 cells were divided into four groups: Untransfection, Lipofectamine, Integrin α5β1 siRNA-transfected group and PD98059 group. The protein expression levels of integrin α5β1 were detected by Western blot analysis and the expression levels of integrin α5β1 mRNA was measured by reverse transcription-polymerase chain reaction (RT-PCR). The protein expression level of ERK1/2, MMP-9 and caspase-3 were measured by Western blot analysis. The proliferation and apoptosis of A549 cells were measured by MT assay and Annexin-V FITC PI double staining.

**Results:**

Integrin α5β1 siRNA could inhibit the phosphorylated ratio of ERK by down-regulate the expression of ERK 1/2 proteins. In addition, integrin α5β1 siRNA or PD98059 could inhibit the proliferation of A549 cells, induce the apoptosis of A549 cells, upregulate the expression of caspase-3 and down-regulate the expression of MMP-9.

**Conclusion:**

Integrin α5β1 might involves the abnormal proliferation and migration of A549 cells through mediating ERK signal transduction pathway.

整合素（integrin）是细胞粘附分子中一类重要的细胞表面受体家族，由α和β两个亚基组成的跨膜异二聚体，α和β亚基均由长的胞外区、跨膜区和短的胞内区组成，主要介导信息从细胞外基质向细胞内传递，调控细胞与胞外基质的粘附和细胞间的粘附，并参与调控细胞的增殖、分化、伸展与迁移等^[1]^。因此整合素的信息传递在促进肿瘤细胞失控制性生长、肿瘤细胞的去分化与远处转移发挥重要作用^[2]^。但是，对于整合素的细胞内外信号传导的具体过程及众多整合素相关蛋白的功能目前仍不十分清楚。整合素α5β1是整合素分子家族中重要的亚单位，能与不同的亚单位结合，构成细胞外基质（extracellular matrix, ECM）的绝大多数受体。近年研究^[3, 4]^显示整合素α5β1的高表达与非小细胞肺癌（non-small cell lung cancer, NSCLC）的转移性、浸润性和低分化趋向密切相关，是NSCLC患者预后不良的危险因素。细胞外信号调节激酶（extracelluar signal-regulated protein kinase, ERK）通路是细胞内重要的信号转导系统，可将胞外刺激信号转导至细胞及其核内，介导细胞生物学反应（如增殖、分化、转化及凋亡等）的过程，与肿瘤的发生发展密切相关^[5]^。整合素α5β1是否通过ERK1/2信号通路调控了肺癌细胞发生发展过程，目前尚无相关研究报道。本实验应用整合素α5/β1 siRNA双链及ERK抑制剂PD98095干预人肺癌细胞A549，旨在研究整合素α5β1蛋白表达对A549细胞生长和迁移能力的影响及其细胞内机制。

## 1 材料与方法

### 1.1 主要试剂与仪器

人肺癌细胞株A549由华中科技大学附属同济医院呼吸内科重点实验室赠送。RPMI-1640培养基购自Hyclone公司，Oligofectamine^TM^和Opti-MEMI购自Carlsbad公司，整合素α5/β1小片段干扰核糖核酸（small interfering RNA, siRNA）双链、兔抗人整合素α5、β1单克隆抗体均购自美国Santa Cruz公司，新生牛血清和Trizol试剂盒购自Gibco公司，Annexin V-FITC凋亡检测试剂盒购自晶美生物工程有限公司，碘化丙锭（propidium iodide, PI）购自Si gma公司，二甲基亚砜及噻唑蓝（methylthiazolyldiphenyl-tetrazolium bromide, MTT）均购自Clontech公司，ERK抑制剂（PD98095）购自美国CST公司，兔抗人磷酸化ERK1/2（p-ERK1/2）单克隆抗体、小鼠抗人ERK1/2单克隆抗体，小鼠抗人基质金属蛋白酶（matrix metalloprotease, MMP）-9单克隆抗体，兔抗人半胱天冬酶（caspase）-3单克隆抗体购自美国R & D公司。TaqDNA聚合酶、莫洛尼(氏)鼠白血病病毒（moloney murine leukemia virus, M-MLV）、寡脱氧胸苷酸、核糖核酸酶抑制剂及琼脂糖均购自美国Promega公司，聚合酶链式反应（polymerase chain reaction, PCR）试剂盒，整合素α5、整合素β1及内参磷酸甘油醛脱氢酶（glyceraldehyde phosphate dehydrogenase, GAPDH）引物购自日本Takara公司，增强型ECL化学发光检测试剂盒购自美国Plierce公司。PCR和蛋白质印迹（Western blot）图像由Bio-Rad公司Gel Doc XR凝胶成像系统扫描，图像分析用Quantity One（version 4.6）分析软件完成。

### 1.2 细胞培养、分组

A549细胞用含10%新生牛血清的RPMI-1640培养基，于37 ℃、5%CO_2_培养箱内培养，0.25%胰蛋白酶消化传代。用瞬时转染方法特异性抑制整合素α5β1表达。利用ERK抑制剂PD98059抑制ERK的磷酸化。取对数生长期A549细胞接种，待细胞融合度达95%时，进行干预。将细胞分为4组：①未转染组：A549细胞不加任何干预；②脂质体组：A549细胞与Oligofectamine脂质体按比例混合；③整合素α5/β1 siRNA转染组：整合素α5和β1 siRNA与Oligofectamine脂质体按比例混合经Opti-MEMI稀释，加入培养好的细胞中，转染步骤按Santa Cruz公司说明书进行。转染培养6 h后，在荧光显微镜下观察，可见荧光素异硫氰酸酯（FITC）标记的siRNA转染的细胞转染成功。然后改为10%血清的RPMI-1640培养基继续培养18 h后应用RT-PCR和Werstern blot法检测RNA干扰效果；④PD98059组：A549细胞加入PD98059 10 μmol/L培养24 h。每组设4个复孔。

### 1.3 RT-PCR和Werstern blot法检测A549细胞中整合素α5β1蛋白和mRNA表达

转染24 h后分别提取总RNA、总蛋白。

首先将RNA逆转录成cDNA，随后PCR扩增。整合素α5基因片段，上游引物序列为5′-TCTGCCTCAATGCTTCTGG-3′，下游引物序列为5′-GTTGAGAGCGATGTGAATCG-3′，扩增片段248 bp。整合素β1基因片段，上游引物序列为5′-ACACGTCTCTCTCTGTCG-3′，下游引物序列为5′-CAGTTGTTACGGCACTCT-3′，扩增片段158 bp。GAPDH作为内参照，上游引物序列为：5'-TCCCATCACCATCTTCCA-3'，下游引物序列为：5'-CATCACGCCACAGTTTCC-3'，扩增产物片段376 bp。PCR反应条件设置如下：94 ℃预变性2 min，94 ℃变性60 s，58 ℃退火45 s，72 ℃延伸1 min，35个循环。各取5 μL PCR产物与2 μL溴酚蓝混合后上样，1.5%琼脂糖凝胶电泳。紫外光下观察并照相，采用软件分析目的基因和GAPDH条带的光密度（*A*）值，以同一管中目的基因和GAPDH产物条带*A*值之比作为反映目的基因mRNA表达水平的数据。

提取细胞总蛋白质后，用BCA蛋白浓度测定试剂盒测定蛋白质浓度以保证每个上样孔总蛋白量一致。蛋白变性、上样，12%十二烷基硫酸钠-聚丙烯酰胺凝胶电泳，随后转膜，孵育一抗（稀释比例1:1, 000）、二抗（稀释比例1:2, 000），ECL发光，胶片曝光。以β-actin作为内参照。胶片扫描并照相，测定各条带灰度值。以目的蛋白和β-actin条带A值之比作为反映目的蛋白表达水平。实验重复3次。

### 1.4 Western blot检测转染对ERK1/2蛋白表达的影响

收集细胞，采用Western blot检测ERK1/2和p-ERK1/2蛋白表达，一抗ERK1/2、p-ERK1/2按1:1, 000稀释，方法同前。曝光后X光片用凝胶图像分析软件扫描，测定蛋白条带的灰度值。结果均用实际值与内参β-actin的比值（*A*%）表示，并计算ERK磷酸化，即p-ERK1/2蛋白占ERK1/2蛋白表达的百分率（%）。每组重复实验3次。

### 1.5 MTT法检测细胞增殖

96孔板内的A549培养结束4 h前，每孔加入20 μL MTT溶液（5 mg/mL），培养4 h后去培养液，加入150 μL二甲基亚砜充分溶解结晶物，采用酶标仪检测490 nm处吸光度*A*值。

### 1.6 Annexin-V FITC PI双染色法检测细胞凋亡

收集细胞，磷酸盐缓冲液漂洗两次，加10 μL Annexin V-FITC和5 μL PI，轻轻混匀，避光室温作用15 min，于流式细胞仪检测早期凋亡细胞百分率（Annexin V-FITC阳性，PI阴性）。

### 1.7 Western blot检测A549细胞中caspase-3、MMP-9蛋白的表达

每孔取20 μg总蛋白加入等体积的上样缓冲液，煮沸变性10 min后，进行制胶、12%聚丙烯酰胺凝胶电泳，泳闭，经电转印将蛋白质转移到硝酸纤维素膜上，封闭后，加入一抗（caspase-3、MMP-9均按1:1, 000稀释），4 ℃孵育过夜，漂洗后加入辣根过氧化物酶标记二抗（1:2, 000稀释），室温摇床上孵育2 h，充分漂洗后按ECL发光试剂盒说明书操作，曝光后X光片用凝胶图像分析软件扫描，测定蛋白条带的灰度值。结果均用实际值与内参β-actin的比值（A%）表示。实验重复3次。

### 1.8 统计学分析

数据使用SPSS 12.0统计软件分析，所有数值以Mean±SD表示。组间数据比较采用单因素方差分析，任意两组均数之间的比较采用*SNK-q*检验。*P* < 0.05为差异有统计学意义。

## 2 结果

### 2.1 整合素α5β1 siRNA导入细胞的证实

经FITC标记的整合素α5β1 siRNA与A549共培养6 h后，细胞质、细胞核均有染色，部分细胞核核仁染色突出，有明亮的荧光（图 1）。

**1:**
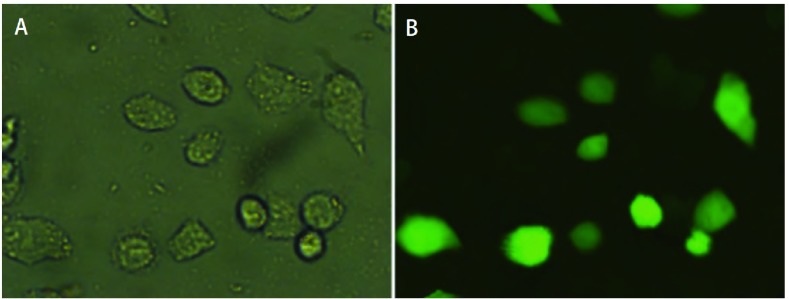
转染后A549细胞 A549 cells were transfected and watched

### 2.2 转染后A549细胞中整合素α5β1蛋白和mRNA表达受抑制

如图 2所示，以β-actin为内参测定各组灰度比值，与脂质体组整合素α5蛋白（0.891±0.063）、整合素β1蛋白（0.963±0.082）比较，转染后整合素α5、β1蛋白表达（分别为0.227±0.071、0.375±0.028）明显降低。脂质体组与未转染组相比差异无统计学意义（*P* > 0.05），提示整合素α5/β1 siRNA双链转染24 h后对整合素α5β1蛋白表达有抑制作用。采用RT-PCR检测是否在整合素α5β1蛋白明显降低的同时伴有整合素α5β1 mRNA的表达变化。整合素α5β1 mRNA的表达与整合素α5β1蛋白表达具有相似的趋势，与脂质体组整合素α5 mRNA（0.78±0.12）、整合素β1 mRNA（0.56±0.09）比较，转染组整合素α5 mRNA、β1 mRNA（分别为0.29±0.03、0.17±0.02）表达均明显降低。脂质体组与未转染组相比差异无统计学意义（*P* > 0.05）。转染对各组细胞中β-actin的蛋白及GAPDH的mRNA表达无任何影响。干扰具有特异性。

**2:**
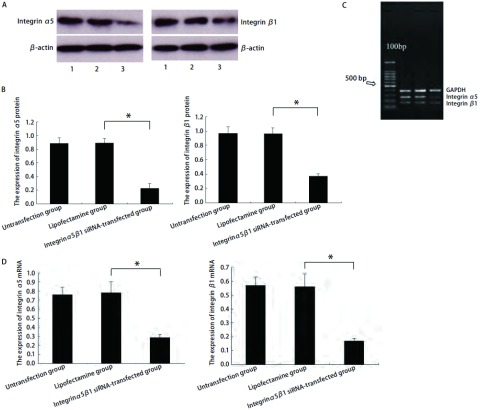
整合素*α*5/*β*1 siRNA抑制A549细胞中整合素*α*5*β*1蛋白和mRNA的表达（1.未转染组；2.脂质体组；3.整合素*α*5/*β*1 siRNA转染组）。A：A549细胞中整合素*α*5*β*1的Western blot检测结果，以*β*-actin作为内参；B：柱状分析图，结果显示整合素*α*5/*β*1 siRNA抑制整合素*α*5*β*1的蛋白表达；C：A549细胞中整合素*α*5*β*1的RT-PCR检测结果，以GAPDH作为内参；D：柱状分析图，结果显示整合素*α*5/*β*1 siRNA抑制整合素*α*5*β*1 mRNA表达。^*^P < 0.05. The inhibition of integrin *α*5*β*1 protein and mRNA in A549 by integrin *α*5*β*1 small interfering RNA (1. Untransfection group; 2. Lipofectamine group; 3. Integrin *α*5*β*1 siRNA-transfected group). A: The expression of integrin *α*5*β*1 protein in A549 detected by Western blot, *β*-actin served as internal control; B: The densitometric analysis of integrin *α*5*β*1 shows the integrin *α*5*β*1 small interfering RNA suppressed the expression of integrin *α*5*β*1 protein; C: The expression of integrin *α*5*β*1 mRNA in A549 detected by RT-PCR, GAPDH served as internal control; D: The densitometric analysis of integrin *α*5*β*1 shows the integrin *α*5*β*1 small interfering RNA suppressed the expression of integrin *α*5*β*1 mRNA. ^*^P < 0.05.

### 2.3 转染后A549细胞中ERK磷酸化受抑制

与脂质体组（分别为0.272±0.015、0.217±0.006）比较，转染整合素α5/β1 siRNA双链24 h后ERK1/2蛋白（0.089±0.017）和p-ERK1/2蛋白（0.042±0.017）表达水平均明显降低，ERK磷酸化水平降低（30.96±3.07）%。脂质体组与未转染组相比差异无统计学意义，提示抑制整合素α5/β1蛋白和mRNA表达的同时可以明显降低ERK磷酸化，提示整合素α5/β1与ERK1/2信号通路之间具有密切关系（图 3）。

**3:**
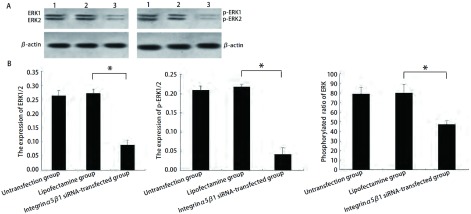
整合素*α*5/*β*1 siRNA抑制A549细胞中ERK磷酸化（1.未转染组；2.脂质体组；3.整合素*α*5/*β*1 siRNA转染组）。A：A549细胞中ERK1/2和p-ERK1/2的Western blot检测结果，以*β*-actin作为内参；B：柱状分析图，结果显示整合素*α*5/*β*1 siRNA可以通过抑制ERK1/2和p-ERK1/2的蛋白表达来抑制ERK磷酸化。^*^P < 0.05。 The inhibition of the phosphorylated ratio of ERK in A549 by integrin *α*5*β*1 small interfering RNA (1. Untransfection group; 2. Lipofectamine group; 3. Integrin *α*5*β*1 siRNA-transfected group). A: The expression of ERK1/2 and p-ERK1/2 in A549 detected by Western blot, *β*-actin served as internal control; B: The densitometric analysis shows the integrin *α*5*β*1 small interfering RNA could suppress the phosphorylated ratio of ERK by down-regulating the expression of ERK 1/2 and p-ERK1/2 proteins. ^*^P < 0.05.

### 2.4 整合素α5/β1介导ERK1/2信号通路抑制A549细胞增殖，促进细胞凋亡

与未转染组和脂质体组比较，转染整合素α5/β1 siRNA双链24 h后A549细胞增殖受到抑制，差异有统计学意义（*P* < 0.01），而在未转染组和脂质体组A549细胞增殖差异无统计学意义。未转染组中细胞增殖可以被ERK抑制剂PD98059所抑制。与未转染组和脂质体组比较，转染整合素α5/β1 siRNA双链24 h后A549早期凋亡细胞百分率明显增高，而在未转染组和脂质体组A549早期凋亡细胞百分率差异无统计学意义。未转染组中早期凋亡细胞百分率同样可以被ERK抑制剂PD98059所提高，提示整合素α5/β1可能介导ERK1/2信号通路抑制A549细胞增殖，促进细胞凋亡（表 1）。

**1:** 整合素*α*5/*β*1 siRNA对A549细胞增殖和凋亡的影响 Effect of integrin *α*5*β*1 small interfering RNA on the proliferation and apoptosis of A549 cells

Group	*A*_490_ value	The percentage of the early apoptotic cells (%)
Untransfection group	1.570±0.047	9.72±0.26
Lipofectamine group	1.531±0.022	9.96±0.13
Integrin *α*5*β*1 siRNA-transfected group	0.499±0.026^*▲^	21.77±0.45^*▲^
PD98059 group	0.541±0.038^*^	17.86±0.29^*^
^*^P < 0.01, significant difference versus Untransfection group; ^▲^*P* < 0.01, significant difference versus Lipofectamine group.		

### 2.5 整合素α5/β1介导ERK1/2信号通路促进caspase-3表达增强

如图 4所示，转染后caspase-3蛋白（1.671±0.027）表达增高，而未转染组（0.869±0.022）和脂质体组（0.851±0.067）比较差异无统计学意义。PD98059组caspase-3蛋白（1.604±0.011）表达也增高。

**4:**
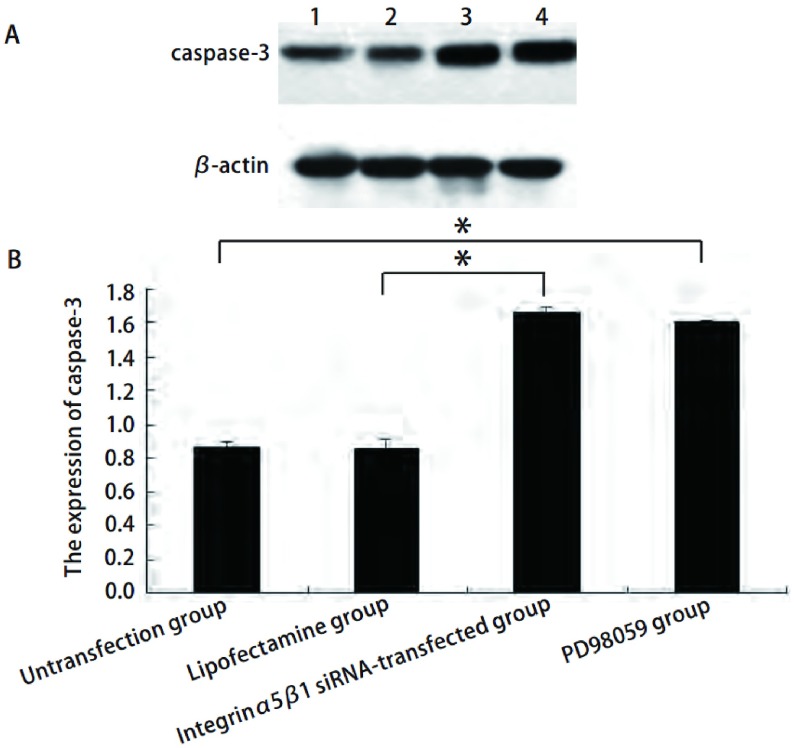
整合素*α*5/*β*1 siRNA对A549细胞中caspase-3表达的影响（1.未转染组；2.脂质体组；3.整合素*α*5/*β*1 siRNA转染组；4. PD98059组）。A：A549细胞中caspase-3的Western blot检测结果，以*β*-actin作为内参；B：柱状分析图，结果显示整合素*α*5/*β*1 siRNA促进caspase-3蛋白表达。^*^P < 0.05。 Effect of integrin *α*5*β*1 small interfering RNA on caspase-3 protein in A549. (1. Untransfection group; 2. Lipofectamine group; 3. Integrin *α*5*β*1 siRNA-transfected group; 4. PD98059 group). A: The expression of caspase-3 in A549 detected by Western blot, *β*-actin served as internal control; B: The densitometric analysis of caspase-3 shows the integrin *α*5*β*1 small interfering RNA could up-regulate the expression of caspase-3. ^*^P < 0.05.

### 2.6 整合素α5/β1介导ERK1/2信号通路抑制MMP-9蛋白的表达

如图 5所示，转染后MMP-9蛋白（0.207±0.020）表达下降，而未转染组（0.912±0.041）和脂质体组（0.920±0.087）比较差异无统计学意义。PD98059组MMP-9蛋白（0.186±0.033）表达也降低。

**5:**
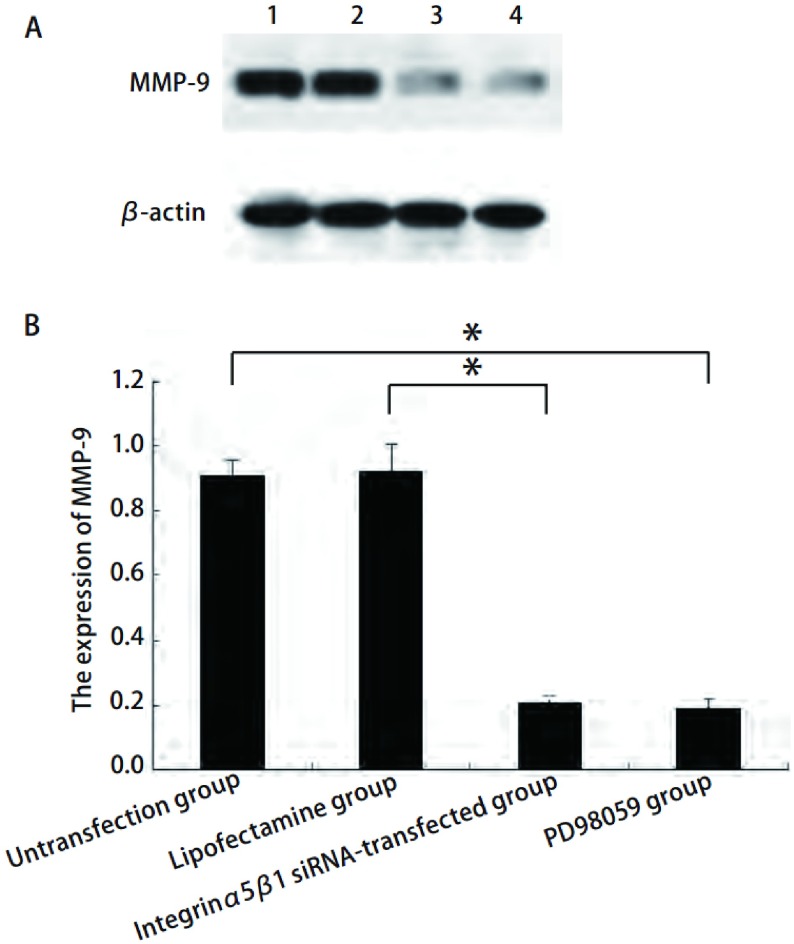
整合素*α*5/*β*1 siRNA对A549细胞中MMP-9蛋白表达的影响（1.未转染组；2.脂质体组；3.整合素*α*5/*β*1 siRNA转染组；4. PD98059组）。A：A549细胞中MMP-9的Western blot检测结果，以*β*-actin作为内参；B：柱状分析图，结果显示整合素*α*5/*β*1 siRNA抑制MMP-9蛋白表达。^*^P < 0.05. Effect of integrin *α*5*β*1 small interfering RNA on MMP-9 protein in A549 (1. Untransfection group; 2. Lipofectamine group; 3. Integrin *α*5*β*1 siRNA-transfected group; 4. PD98059 group). A: The expression of MMP-9 in A549 detected by Western blot, *β*-actin served as internal control; B: The densitometric analysis of MMP-9 shows the integrin *α*5*β*1 small interfering RNA could suppress the expression of MMP-9. ^*^P < 0.05.

## 3 讨论

整合素是由α和β两条链以共价键连接构成的细胞表面受体家族，以异二聚体的形式表达于细胞表面.不仅可以通过识别细胞外基质的精氨酸-甘氨酸-天冬氨酸（Arg-Gly-Asp, RGD）序列介导细胞与细胞外基质之间的粘附，与免疫球蛋白超家族分子结合介导细胞与细胞间的粘附，还可以双向转导细胞内外的信号，直接影响细胞的生存、生长、增殖、分化以及肿瘤细胞的侵袭和转移等生物学行为。

Adachi等^[3]^的研究显示NSCLC组织中整合素α5β1的高表达与肿瘤分级负相关，低分化NSCLC中的整合素α5β1表达明显高于高分化NSCLC，且整合素α5β1高表达者的生存期明显低于表达正常者。Oshita等^[4]^的研究也表明在NSCLC组织中，整合素α5β1和p53是影响预后的最主要危险因素。因此，整合素α5β1的高表达可能在NSCLC生长、增殖、凋亡和转移的调控中起到重要作用。本组研究结果表明，转染整合素α5/β1 siRNA双链后A549细胞增殖受到抑制，早期凋亡百分率增加，MMP-9蛋白表达下降提示整合素α5β1参与调控肺癌A549细胞凋亡、增殖、侵袭和转移，整合素α5/β1 siRNA双链通过抑制整合素α5/β1蛋白和mRNA表达抑制肺癌A549细胞生长和迁移。

广泛存在于肿瘤细胞表面的整合素受体及其引发的异常信号转导机制在肿瘤的转移、浸润及细胞的增殖和凋亡中起着重要作用^[6, 7]^。以整合素为靶点的药物也正在进行^[8]^。整合素α5β1是整合素分子家族中重要的亚单位，在肺癌细胞的去分化与远处转移中发挥重要作用^[9]^。然而在整合素活性调节机制中，还有很多没有阐明的问题，如整合素介导的由外而内的信号传递和由内而外的信号传递之间有什么相互关系？细胞通过哪些通路和分子来实现对整合素活性的调控？为此，我们选用整合素α5/β1 siRNA双链和ERK抑制剂PD98095干预人肺癌细胞A549，旨在探讨整合素α5/β1是否介导了ERK1/2信号通路影响A549细胞生长和迁移。研究显示，转染整合素α5/β1siRNA双链后A549细胞的ERK磷酸化明显受到抑制，提示整合素α5β1与ERK1/2信号通路密切相关，进一步研究显示ERK抑制剂PD98059同样能抑制A549细胞增殖，促进早期凋亡和抑制MMP-9蛋白表达，提示ERK1/2信号通路同样参与调控了肺癌A549细胞凋亡、增殖、侵袭转移，整合素α5β1可能介导ERK1/2信号通路调控A549细胞的生长与迁移。国外研究^[10, 11]^显示整合素α5β1的高表达状态与慢性髓细胞白血病（chronic myeloid leukemia, CML）的发病息息相关，通过FAK-MAPK或PI3K-AKT信号途径上调核内某些基因的表达，促使细胞发生白血病转化、异常增殖和凋亡受抑。有研究^[9, 12]^显示整合素β1通过ERK1/2信号通路上游的FAK调控肺癌细胞的增殖。α-倒捻子素可以抑制由佛波酯诱导活化的整合素α3后通过FAK-ERK-NF-κB信号途径调控MMP-2和MMP-9的表达，从而抑制A549细胞迁移^[13]^。骨桥蛋白可以识别整合素αvβ3，启动FAK-PI3K-ERK细胞内信号通路，促进A549细胞的迁移^[14]^。因此，ERK1/2信号通路在调控A549细胞的生长、增殖、凋亡和转移中具有重要作用，阻断这些信号通路上游的整合素，其介导的生长、增殖、凋亡和转移必然受到影响。

此外，我们同时亦发现整合素α5/β1 siRNA双链及ERK抑制剂PD98059均可以使A549细胞中caspase-3表达增高，提示整合素α5β1可能介导了ERK1/2信号通路诱导caspase-3表达增高，从而促使凋亡的发生，阻断整合素α5β1有可能防止细胞恶性转化、肿瘤侵袭转移以及耐药的发生。已有研究^[15]^显示整合素α5β1拮抗剂在胶质母细胞瘤细胞实验治疗中有明显促进细胞凋亡、诱导细胞老化的作用。

总之，我们的研究证实，在A549细胞中整合素α5β1可能通过诱导ERK的磷酸化，进而抑制caspase-3蛋白和促进MMP-9蛋白的表达，从而发挥调控细胞的生长与迁移作用。本研究揭示了整合素α5β1在A549细胞中的作用及相关细胞内信号调节机制，为以整合素为靶点的肺癌分子治疗提供了实验基础和理论依据。
